# Roles, Functions, and Pathological Implications of Exosomes in the Central Nervous System

**DOI:** 10.3390/ijms26031345

**Published:** 2025-02-05

**Authors:** Sonia Spinelli, Domenico Tripodi, Nicole Corti, Elena Zocchi, Maurizio Bruschi, Valerio Leoni, Roberto Dominici

**Affiliations:** 1 Laboratory of Molecular Nephrology, IRCCS Istituto Giannina Gaslini, 16147 Genoa, Italy; soniaspinelli@gaslini.org (S.S.); mauriziobruschi@gaslini.org (M.B.); 2 Laboratory of Clinical Pathology and Toxicology, Hospital Pio XI of Desio, ASST-Brianza, 20832 Desio, Italy; domenicotripodi96@gmail.com (D.T.); nicole.corti@asst-brianza.it (N.C.); roberto.dominici@asst-brianza.it (R.D.); 3 Department of Experimental Medicine (DIMES), University of Genoa, 16132 Genoa, Italy; ezocchi@unige.it; 4 Department of Medicine and Surgery, University of Milano-Bicocca, 20900 Monza, Italy

**Keywords:** extracellular vesicles (EVs), exosomes, neurodegenerative diseases, blood–brain barrier, peripheral–brain axis, therapeutic delivery

## Abstract

Exosomes are a subset of extracellular vesicles (EVs) secreted by nearly all cell types and have emerged as a novel mechanism for intercellular communication within the central nervous system (CNS). These vesicles facilitate the transport of proteins, nucleic acids, lipids, and metabolites between neurons and glial cells, playing a pivotal role in CNS development and the maintenance of homeostasis. Current evidence indicates that exosomes from CNS cells may function as either inhibitors or enhancers in the onset and progression of neurological disorders. Furthermore, exosomes have been found to transport disease-related molecules across the blood–brain barrier, enabling their detection in peripheral blood. This distinctive property positions exosomes as promising diagnostic biomarkers for neurological conditions. Additionally, a growing body of research suggests that exosomes derived from mesenchymal stem cells exhibit reparative effects in the context of neurological disorders. This review provides a concise overview of the functions of exosomes in both physiological and pathological states, with particular emphasis on their emerging roles as potential diagnostic biomarkers and therapeutic agents in the treatment of neurological diseases.

## 1. Introduction

Neurological disorders are debilitating conditions that impact the nervous system, primarily including acute central nervous system (CNS) injuries such as spinal cord injury (SCI), traumatic brain injury (TBI), and stroke, as well as neurodegenerative diseases like Alzheimer’s Disease (AD) and Parkinson’s Disease (PD) [1,2]. The diagnosis of acute CNS injuries relies mainly on clinical presentations and imaging modalities, including computed tomography (CT), magnetic resonance imaging (MRI), and X-ray [3]. For acute CNS injuries, surgical intervention remains a critical emergency measure, although no curative reparative strategies exist [4]. Additionally, cerebrospinal fluid (CSF) collection and positron emission tomography (PET) imaging are recommended for diagnosing neurodegenerative diseases [4]. However, the high economic burden limits the clinical application of PET, while CSF punctures carry risks of surgical complications [5]. Furthermore, the lack of specific early symptoms in neurodegenerative diseases results in most patients being diagnosed at mid-to-late stages, where treatment primarily aims to slow disease progression [6]. Thus, the need for more precise and effective methods for diagnosing and treating neurodegenerative diseases arises. Extracellular vesicles (EVs) are subcellular structures secreted via cellular paracrine mechanisms and include apoptotic bodies, microvesicles, and exosomes [7]. Among EVs, exosomes, characterized by their small size (<200 nm) and lipid bilayer enclosure, are particularly significant in mediating communication between neurons and glial cells [8,9]. Exosomes derived from CNS cells can cross the blood–brain barrier (BBB) and transport disease-related molecules, making them detectable in patients’ blood samples [10]. This attribute positions exosomes as promising biomarkers for the diagnosis of neurological disorders. Extensive research has demonstrated the reparative effects of mesenchymal stem cell (MSC)-derived exosomes in various animal models of neurological diseases [11], underscoring their potential as therapeutic agents in clinical settings. This review synthesizes the current understanding of exosomes as mediators of intercellular communication under both physiological and pathological conditions. Additionally, it highlights recent studies on the diagnostic potential of exosomes in neurodegenerative diseases and discusses the prospects of stem cell-derived exosomes as therapeutic tools for neurological disorders.

## 2. Production, Release, Internalization, and Composition of Exosomes

Exosomes perform their roles by either fusing with the plasma membranes of recipient cells or interacting with surface membrane proteins. They collect bioactive molecules such as proteins, nucleic acids (including mRNA, miRNA, lncRNA, etc.), and lipids, serving as mediators of intercellular communication between donor and recipient cells [12]. Among these, miRNA represents a pivotal communication agent within exosomes, capable of modulating gene and protein expression in recipient cells while also preventing exosomal degradation [13,14]. Upon entry into target cells, exosomal miRNA interacts with mRNA of specific target genes via partial sequence complementarity, participating in several processes such as tissue regeneration, inflammation, apoptosis, and other regulatory pathways [15,16]. Since virtually all cell types can release exosomes, they are produced by a wide array of cellular sources (for example, skin, lung, craniofacial, tendon, and renal tissues). The exosomes released in physiological conditions are much different from the ones released in pathological conditions, even within the same cell type [17]. In some circumstances when exosomes fail to interact promptly with their target cells, they are rapidly cleared from blood through metabolic pathways. When applied locally or administered systemically, exosomes offer various therapeutic advantages, such as promoting the repair of damaged intervertebral disks and bone tissues [18].

**Figure 1:**
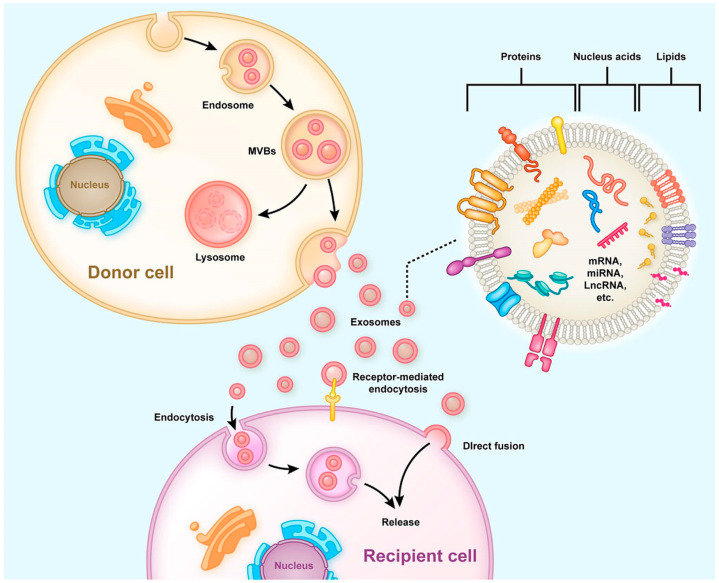
The typical process of exosome nomenclature, classification, biogenesis, secretion, and transfer from donor cells to recipient cells, as well as the structural characteristics of exosomes, is illustrated. Early endosomes initially form intraluminal vesicles (ILVs), which later mature into multivesicular bodies (MVBs). These MVBs then release exosomes into the extracellular space. Exosomes have a bilayer membrane structure containing functional proteins, nucleic acids, and lipids. Some of these components are transferred to recipient cells, where they regulate gene expression and cell function. Reprinted/adapted from ref. [19].

EVs are subcellular entities released via the paracrine pathways of cells. These vesicles are classified primarily into apoptotic bodies (ranging from 500 to 2000 nm in diameter), microvesicles (200 to 2000 nm in diameter), and exosomes (30 to 200 nm in diameter) [7]. Exosomes are characterized as small lipid bilayer-enclosed EVs [8], playing a crucial role in facilitating communication between neurons and glial cells [9]. Furthermore, exosomes derived from cells of the central nervous system (CNS) can transport disease-related molecules across the blood–brain barrier (BBB), making them detectable in the bloodstream of patients [10]. This feature highlights their potential as valuable diagnostic biomarkers for neurological diseases. Moreover, a growing body of research has demonstrated that exosomes derived from mesenchymal stem cells (MSCs) exhibit significant therapeutic effects in treating neurological disorders in various animal models [11], positioning exosomes as promising candidates for future clinical therapies (Figure 1).

**Figure 2:**
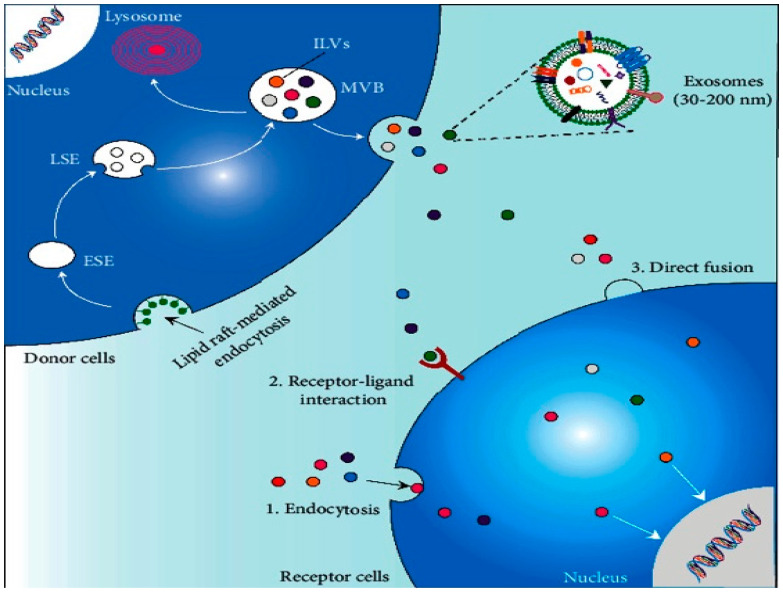
The biosynthesis, secretion, cellular uptake, and molecular composition of exosomes are outlined. Exosome biosynthesis starts with an endosomic pathway. The cytoplasmic membrane invaginates to create early sorting endosomes (ESEs), which mature into late sorting endosomes (LSEs). The membrane of LSEs further invaginates, forming multivesicular bodies (MVBs) that contain numerous intraluminal vesicles (ILVs). MVBs can either fuse with the plasma membrane to release ILVs as exosomes or fuse with lysosomes for degradation. Exosomes deliver specific proteins, nucleic acids, lipids, and metabolites to recipient cells through endocytosis, receptor–ligand interactions, and direct membrane fusion. Reprinted/adapted from ref. [29].

**Figure 3:**
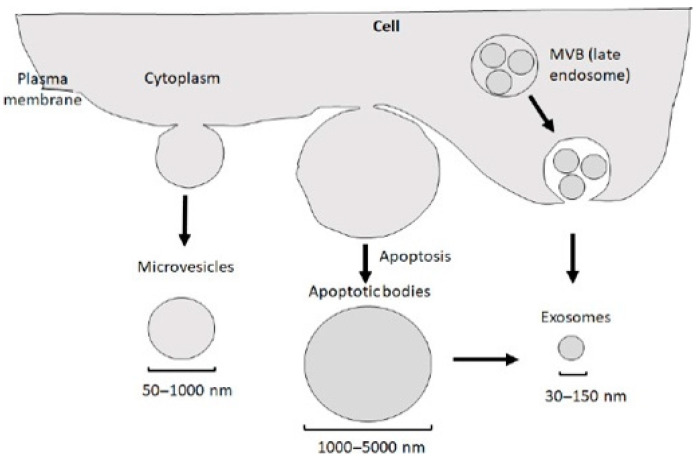
There are three primary types of extracellular vesicles: exosomes, microvesicles, and apoptotic bodies. Each type differs in size, biogenesis, and physiological function. Extracellular vesicles can be classified into three subtypes based on their size and biogenesis: apoptotic bodies (1000–5000 nm), which are formed during apoptosis; microvesicles (50–1000 nm); and exosomes (30–150 nm). It is important to note that, in addition to being directly produced from a cell via the endocytic pathway, exosomes can also be derived from apoptotic bodies. MVB refers to a multivesicular body. Reprinted/adapted from ref. [30].

The biosynthesis of exosomes is a well-regulated process comprising three primary stages: endocytosis, the formation of multivesicular bodies (MVBs), and exosome secretion [20] (Figure 2). During endocytosis, early sorting endosomes (ESEs) mature into late sorting endosomes (LSEs), and the invagination of the LSE membrane generates MVBs containing numerous intraluminal vesicles (ILVs) [21]. The formation of MVBs can be linked to the physical properties and lipid composition of the endosomal microdomain rafts or may be related to cargo sorting, which can occur via the endosomal sorting complex required for transport (ESCRT)-dependent and -independent pathways [22,23]. Once matured, MVBs either fuse with the plasma membrane to release exosomes or are degraded via fusion with lysosomes [17]. The movement of MVBs toward the plasma membrane is mediated by molecular motor proteins, including kinesins. A critical event in exosome secretion involves the docking of MVBs with SNARE proteins located on the plasma membrane [24]. This process is orchestrated by several members of the RAB protein family (RAB7, RAB11, RAB27, and RAB35) [25], alongside RAL-1, a member of the Ral guanosine triphosphatase (GTPase) family [26]. While much has been discovered, the precise mechanisms and regulatory pathways governing exosome biosynthesis and secretion remain under investigation. Once released into the extracellular space, exosomes can exert influence on neighboring cells within the extracellular matrix. Exosome-mediated cargo transfer occurs via three principal mechanisms: endocytosis, direct fusion with the plasma membrane, or receptor–ligand interactions [17]. Exosomes contain a variety of molecular components, including proteins, lipids, nucleic acids, and metabolites. The specific composition of an exosome reflects not only the cellular origin but also the physiological and pathological conditions of the surrounding microenvironment [27]. A database was recently generated to facilitate exosomal research for the bioinformatics analysis of exosomal “cargo” [28] (Figure 2 and Figure 3).

## 3. Exosome Extraction, Detection, and Characterization

Exosomes can be isolated from various biological fluids and cellular sources. Based on the physical and chemical properties of exosomes, several extraction techniques have been developed, allowing researchers to select appropriate separation methods in accordance with their purity requirements. Common isolation techniques include ultracentrifugation [31], size exclusion chromatography [32], immune-affinity capture [33], ultrafiltration [34], commercial kits [35], and microfluidics [36]. Each of these methods possesses distinct advantages, limitations, and application ranges, as summarized in Table 1. Among these, the ultracentrifugation is considered the gold standard for exosome extraction and is widely utilized in research settings [37]. However, this isolation technique requires expensive equipment and is labor-intensive, raising concerns regarding the integrity and biological activity of the isolated exosomes, thereby limiting its applicability in clinical diagnostics [38]. At present, no single method exists that consistently yields exosomes with high yield, high purity, and optimal integrity to fulfill the diverse requirements of various applications. Thus, researchers must select different extraction methods or combinations thereof based on their specific experimental needs. Exosomes can be stored and maintained using several methods. Commonly, they are preserved by freezing at −80 °C or in liquid nitrogen to prevent degradation over time. For short-term storage, exosomes are often kept at 4 °C. Additionally, some studies use cryoprotectants to protect the exosomes during freezing. Another approach is lyophilization (freeze drying), which allows for long-term storage at room temperature. Finally, exosome integrity can be maintained by avoiding repeated freeze–thaw cycles, which can lead to the loss of their functionality [10]. The isolated exosomes can be detected and characterized according to their biochemical properties (Table 1). The morphology and particle size of exosomes are typically analyzed using scanning electron microscopy [39] and transmission electron microscopy. Dynamic light scattering, a nanoparticle tracking analysis, and tunable resistive pulse sensing are frequently employed for the rapid assessment of exosome particle size and concentration [40]. The biological functions of exosomes primarily depend on their content. Traditional techniques for protein detection in exosomes predominantly include Western blotting (WB) and enzyme-linked immunosorbent assays (ELISAs), while high-throughput sequencing and PCR amplification are utilized for the detection of exosomal RNA [8]. Furthermore, novel methodologies such as microfluidic chips [41], droplet digital PCR [42], and ion-exchange nanomembrane detection [43] have also been adopted to characterize exosomes. The group coordinated by Maja Mustapic from NIA pioneered the identification of extracellular EVs enriched for a neuronal origin from peripheral blood and using them as a biomarker discovery platform for neurological disorders. Their approach turned out to be better, in terms of sensitivity and accuracy for biomarker discovery, than serum, plasma, or non-enriched total plasma EVs [44]. Exosomes play a crucial role in intercellular communication by transporting proteins, lipids, and RNA molecules. Their specificity and “tropism” are influenced by surface proteins, lipids, and the molecular cargo they carry. In animal and human studies, exosomes have been shown to preferentially target certain cell types, often due to interactions between surface molecules, such as integrins, and receptors on recipient cells. This specificity is critical for applications like drug delivery and cancer therapy, as exosomes can be engineered to target tumor cells or immune cells.

However, the mechanisms behind exosome tropism are not fully understood, and ongoing research aims to elucidate how their cargo and surface characteristics determine targeting efficiency. Understanding exosome tropism is essential for optimizing their use in therapeutic applications.

## 4. Functions of Exosomes as Mediators of Intercellular Communication in Physiological and Pathological Contexts in the CNS

**Figure 4:**
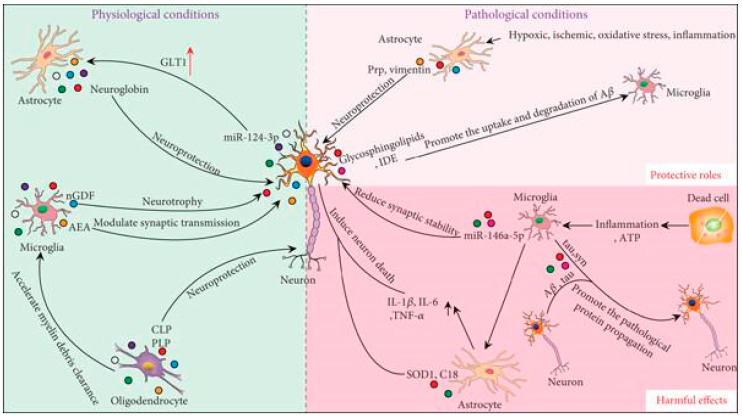
Functions of exosomes as mediators of intercellular communication in both physiological and pathological contexts. Under both physiological and pathological conditions, cells can deliver substances and exchange information in multiple directions through exosomes. The following substances are commonly associated with exosome content: nGDF (nervous growth/differentiation factor), AEA (N-arachidonoylethanolamine), PLP (proteolipid protein), CNP (2′3′-cyclic-nucleotide-phosphodiesterase), PrP (prion protein), Aβ (amyloid β), IDE (insulin-degrading enzyme), and SOD1 (mutant superoxide dismutase). Reprinted/adapted from ref. [29].

Exosomes, secreted by neurons, astrocytes, microglia, and oligodendrocytes, play a crucial role in intercellular communication (Figure 4). Under normal physiological conditions, interactions between neurons and glial cells are essential for maintaining homeostasis and supporting the development of the CNS [45]. Neuronal exosomes facilitate the transmission of signaling information, regulating the formation and maturation of neural circuits [46]. Additionally, their content reflects the cellular environment, whether physiological or pathological. Exosomes released by cultured cortical neurons and astrocytes may regulate synaptic activity and enable the exchange of membrane proteins within the brain. They also act as neuron-specific carriers to glial cells, modulating their functions [47]. For instance, neuronal exosomes can deliver miR-124-3p to astrocytes, upregulating the glutamate transporter GLT1 in these cells [48]. Similarly, astrocyte-derived exosomes can transfer neuroglobin to neurons, offering protection against cellular stress [49]. Microglial exosomes transport neurotrophic factors, such as nerve growth/differentiation factor (nGDF) to neurons, promoting neuronal survival and function [50]. They may also carry N-arachidonoylethanolamine (AEA), targeting GABAergic neurons to modulate synaptic transmission [51]. Microglia can internalize exosomes from oligodendrocytes to assist in clearing myelin debris [52]. Exosomes from oligodendrocytes contain significant levels of myelin proteolipid protein (PLP) and 2′,3′-cyclic-nucleotide-phosphodiesterase (CNP), which support neuronal functions and maintain myelin protein–lipid balance [53]. Under pathological conditions, such as ischemia, hypoxia, oxidative stress, and inflammation, exosomes can have either protective or harmful effects [54]. On the protective side, they support neuronal survival and help clear pathological proteins. For example, astrocyte-derived exosomes carrying prion protein (PrP) enhance neuronal resilience to hypoxic and ischemic stress [55]. Following spinal cord injury (SCI), astrocytes release vimentin through exosomes, providing neuroprotection [56]. In Alzheimer’s Disease (AD), characterized by amyloid β (Aβ) and hyperphosphorylated tau accumulation, neuronal exosomes rich in glycosphingolipids can sequester extracellular Aβ, promoting its uptake and degradation by microglia [57,58]. An exosomal insulin-degrading enzyme (IDE) also encourages microglial clearance of Aβ [59]. Conversely, exosomes can exacerbate disease progression by spreading inflammation and pathological proteins within the CNS. For instance, pro-inflammatory microglia transfer exosomes to astrocytes, activating neurotoxic A1 astrocytes that harm neurons [60,61]. ATP released by necrotic cells attracts microglia to injury sites, altering the proteome of microglial exosomes, which subsequently activate astrocytes and upregulate pro-inflammatory cytokines such as IL-1β, IL-6, and TNF-α [62]. Astrocyte-derived exosomes can also transfer mutant superoxide dismutase (SOD1), pro-apoptotic C18 ceramide, and complement proteins to neurons, contributing to neuronal damage [63,64,65]. Exosomes are implicated in the propagation of toxic Aβ and tau between neurons, furthering neuronal death and the spread of pathological proteins [66,67]. Microglia can secrete exosomes that facilitate tau transmission [68]. In Parkinson’s Disease (PD), exosomes create a favorable environment for the aggregation of α-synuclein (α-syn) [69]. Microglial exosomes also promote α-syn propagation and neuronal death [70,71]. MicroRNAs (miRNAs) within exosomes contribute to the pathogenesis of neurological disorders. For example, miR-15 suppresses beta-secretase 1 (BACE1) expression and tau phosphorylation; yet, its levels are significantly reduced in circulating exosomes of AD patients, suggesting a link between exosomal miRNAs and disease progression [72]. In PD models, serum exosomes show increased miR-137, which induces oxidative stress in neurons [73]. Microglia-derived exosomes carrying miR-146a-5p reduce dendritic spine density and synaptic stability in neurons by inhibiting presynaptic synaptotagmin 1 (Syt1) and postsynaptic neuroligin 1 (Nlg1) [74]. In conclusion, as summarized in Table 2, exosomes mediate multidirectional substance exchange and communication within the CNS, influencing the development and progression of neurological disorders. A thorough understanding of their roles is essential for identifying therapeutic targets and diagnostic tools. Further research is needed to discern the beneficial and detrimental components of exosomes, enabling the design of disease-suppressing exosomal therapies [54]. The different behavior of exosomes in physiological status versus diseases reflects the fact that in conditions of brain pathologies, pathogenetic mechanisms are activated, which lead to an increased generation of molecularly and functionally altered exosomes with the production of modified or aberrant content, such as augmented delivery of pathogenic foreign proteins, coding/non-coding elements, and switching biologically inert exosomes to active exosomes. Notably, exosomes from CNS cells can cross the blood–brain barrier (BBB) and enter the bloodstream, carrying content that reflects the pathological state of their source cells. This property has positioned exosomes as promising biomarkers for diagnosing neurodegenerative diseases.

## 5. Possible Diagnostic Biomarkers of Exosomes in Neurodegenerative Disorders

**Figure 5:**
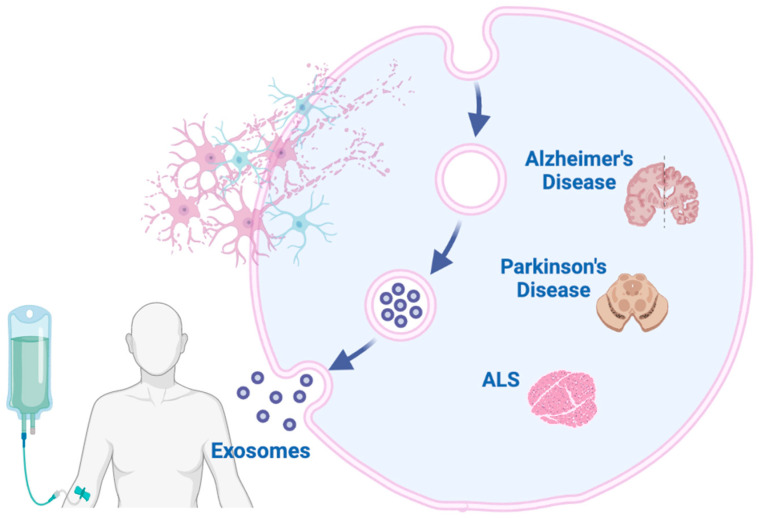
Possible exosomal biomarkers for diagnosing neurodegenerative disorders. Exosomes derived from various brain and peripheral cells may carry disease-specific proteins, nucleic acids, and lipids, which can serve as biomarkers for the early detection and monitoring of neurodegenerative diseases (Alzheimer’s Disease, Parkinson’s Disease, and Amyotrophic Lateral Sclerosis). Their presence and composition in biological fluids, such as blood and cerebrospinal fluid, offer promising avenues for non-invasive diagnostic approaches.

Neurodegenerative diseases are characterized by chronic and progressive pathology, often resulting in irreversible damage. By the time patients exhibit symptoms and seek medical attention, these diseases have typically advanced to the middle or late stages, where available treatments offer only limited benefit in slowing disease progression and cannot reverse neuronal damage [74]. Therefore, identifying early diagnostic biomarkers for neurodegenerative diseases is crucial. Since neurodegenerative diseases often present with overlapping clinical symptoms, the identification of disease-specific biomarkers is particularly important. CSF is in direct contact with the brain and spinal cord: in such a way, it can reflect pathological changes occurring within the CNS [75]. However, collecting CSF is not always feasible due to its invasive nature, as lumbar puncture can lead to surgical trauma. Thus, peripheral blood is considered a more accessible alternative for diagnosing neurodegenerative diseases when compared to CSF [76]. The BBB, which is composed of tight junctions between brain endothelial cells, pericytes, astrocytic end-feet, and the capillary basement membrane, acts as a selective barrier between the bloodstream and neuronal tissue. It serves to prevent harmful substances from crossing into the CNS while allowing water and essential metabolites, such as glucose and amino acids, to pass through to maintain CNS function [77,78]. Although the exact mechanism of exosomal entry into the brain is not fully understood, exosomes, due to their unique lipid and protein composition, can cross the BBB with relative ease. Three types of exosomes have been isolated from the human brain: L1CAM of neuronal origin, TMEM119 of microglial origin, and GLAST derived from astrocytes. The exosomes originating from CNS cells can transfer neurodegenerative disease-related molecules across the BBB, making their detection possible in peripheral blood samples (Figure 3). Zhao and colleagues demonstrated that levels of Parkinson disease protein 7 (DJ-1) and α-synuclein from neuron-derived exosomes are significantly higher in the plasma of PD patients compared to healthy controls [79]. Recent studies have shown that exosomes from neuronal origin can represent a novel potential biomarker for AD and frontotemporal dementia (FTD). It has been reported that the concentrations of Aβ and tau proteins in neuronal exosomes from the blood of AD patients are elevated and strongly correlated with CSF levels [80]. Some commercial kits with immunoprecipitation are available to isolate neuron-derived exosomes from blood, with antibodies targeting neural cell adhesion molecules (NCAMs) and L1CAM used to enrich the neuronal exosome population. In 2004, Graca Raposo et al. showed, based on different laboratory techniques, that both forms of prionic protein (PrPc and PrPsc in the culture medium of infected and noninfected cell cultures) are associated with exosomes, indicating the possibility that they may provide for intercellular carriers of both prion PrPc and PrPsc and their spread throughout the organism [81,82]. All together, these studies indicate that exosomes have significant potential as diagnostic biomarkers for neurodegenerative diseases. In fact, exosomal biomarkers may appear in the bloodstream even before clinical symptoms manifest, highlighting the importance of identifying highly sensitive biomarkers for early screening and diagnoses. Exosome-based diagnostic platforms are in a promising stage of clinical translation, although further research is necessary to bridge the gap between the initial discovery of exosomal biomarkers and their application in clinical practice (Figure 5).

### 5.1. Alzheimer’s Disease

Alzheimer’s Disease (AD) is the most prevalent form of dementia, projected to affect approximately 14 million people in the U.S. by 2050. The hallmarks of the disease are the accumulation of extracellular Aβ and intracellular hyperphosphorylated tau proteins. Current treatments offer only symptomatic relief, and no therapies exist that halt or reverse disease progression. There are two pathways for the cleavage of amyloid precursor protein (APP). In the non-amyloidogenic pathway, APP is cleaved by α- and γ-secretase, generating neuroprotective fragments. In the amyloidogenic pathway, β- and γ-secretase cleave APP, producing Aβ peptides of varying lengths [83]. Rajendran et al. demonstrated that APP processing occurs in early endosomes, resulting in the production of Aβ peptides in multivesicular bodies, which are then released via exosomes. They also showed that exosomal markers are enriched in amyloid plaques from the human brain. Exosomes derived from AD patients’ brains are enriched in APP C-terminal fragments, BACE-1, γ-secretase, soluble APPβ, APPα, and Aβ-42 [84]. Another significant result was reported by P. Joshi, who demonstrated that microglia-derived microvesicles (MVs) promote neurotoxicity, as shown in an in vitro analysis, contributing to AD degeneration and that the mechanism responsible results from the capability of MV lipids to promote the formation of soluble Aβ species from extracellular insoluble aggregates and from the presence of neurotoxic Aβ forms trafficked to MVs after Aβ internalization into microglia [85]. Jia et al. found that the levels of growth-associated protein 43 (GAP43), neurogranin, synaptosome-associated protein 25 (SNAP25), and synaptotagmin 1 in neuron-derived exosomes were significantly lower in the blood of AD patients, suggesting their potential use in predicting AD at pre-symptomatic stages [86]. Similar results were obtained by EJ Goetzl, who observed a reduction in synaptic proteins (synaptopodin, synaptotagmin, and synaptophysin) in neuronal exosomes. This finding appears to correlate with disease severity, as these proteins are associated with cognitive performance in AD and FTD [87]. Exosomal Aβ levels, especially oligomeric Aβ, have been found to increase in AD brain samples. These exosomes facilitate the propagation of oligomeric Aβ between neurons [66,88]. Furthermore, proteins such as Alix and flotillin-1, involved in exosome formation, have been detected surrounding neuritic plaques [83]. This suggests that exosomes contribute to the spread of Aβ within the brain, although it remains unclear whether their release is a protective cellular response. The tau protein, which becomes hyperphosphorylated in AD, can also propagate through exosomes. Exosomes isolated from patients with mild cognitive impairment or AD stages injected into mice resulted in increased phosphorylated tau [89]. Studies of post mortem AD brain samples reveal that AD-derived exosomes contain more oligomeric tau than controls, and these exosomes were more efficiently taken up by neurons, propagating tau pathology in wild-type mice [90]. Tau pathology and propagation were reduced when exosome secretion was inhibited, indicating that exosomes are involved in mediating tau spread [91]. Additionally, microglia show a higher efficiency in phagocytosing tau than neurons or astrocytes, and exosomes play a role in this process [68]. Injecting exosomes containing tau from human cells with tau mutations into wild-type mouse brains caused tau inclusions two months post-injection [92]. These data support the role of exosomes in propagating tau in AD. Pathogenic proteins were extracted from neutrally derived blood EVs and quantified to develop biomarkers for the staging of sporadic disease. The numbers of markers such as Aβ1–42, phospho-T181-tau, and phospho-S396-tau are correlated with AD progression and could predict the future disease up to 10 yrs before clinical onset. Additionally, the exosomal protein levels accurately predict the conversion of mild cognitive impairment to AD [89]. In a study published by Kapogiannis’s group, it was shown that some transcription factors quantified in plasma-derived neural EVs from AD were diminished 2–10 years before the clinical diagnosis of disease compared to matched controls [93]. Data from the same group showed that levels of autolysosomal proteins distinguish patients with AD from case controls and appear to reflect the pathology up to 10 years before clinical onset [94,95]. Various preclinical and clinical studies conducted 40 years ago revealed that cell therapy is the only rational and feasible strategy to regenerate neural tissues, mainly using mesenchymal stem cells (MSCs). However, stem cell therapy has disadvantages as its therapeutic molecules cannot pass through the BBB effectively and are detrimental to patient safety. To overcome these limitations, several clinical studies have been conducted based on small extracellular vesicles (sEVs) extracted from MSCs and enriched with miRNAs to treat neurodegenerative diseases more safely and effectively. In AD patients, certain microRNAs (miRNAs), including miR-15, miR-185-5p, and miR-342-3p, are downregulated in serum exosomes, while their predicted target, amyloid precursor protein (APP), is abnormally elevated in the AD brain. This miRNA-mRNA interaction leads to fewer miRNAs being sorted into exosomes [72,96]. Cheng et al. identified sixteen AD-specific miRNAs (miR-101-3p, miR-106a-5p, miR-106b-5p, miR-1306-5p, miR-143-3p, miR-15a-5p, miR-15b-3p, miR-18b-5p, miR-20a-5p, miR-30e-5p, miR-335-5p, miR-342-3p, miR-361-5p, miR-424-5p, miR-582-5p, miR-93-5p) in serum exosomes as potential diagnostic and prognostic biomarkers, using high-throughput next-generation sequencing and qRT-PCR to differentiate between AD patients and healthy individuals. Compared to healthy controls, miR-342-3p, miR-15b-3p, and miR-1306-5p were downregulated in AD patients’ serum exosomes, whereas the other 13 miRNAs were upregulated [97]. It is noteworthy that recent research demonstrated the high expression of miR-384 in AD patients’ serum exosomes, showing high specificity for distinguishing AD from other conditions like vascular dementia (VAD) and Parkinson’s Disease dementia (PDD) [98]. Finally, human MSC-derived exosomes, administered intranasally in a transgenic mouse AD model (5xFAD) exhibited immunoprotective and immunomodulatory abilities reducing behavioral symptoms and Aβ plaque load in AD mouse models, slowing down AD pathogenesis [68,99,100].

### 5.2. Parkinson’s Disease

Parkinson’s Disease (PD) is the second most common neurodegenerative disorder, projected to affect 12 million people by 2040 [101]. PD is marked by the degeneration of dopamine-producing neurons and the accumulation of Lewy bodies, which contain α-synuclein. Current treatments for PD are symptomatic, with no therapies capable of halting neuronal degeneration [102]. Exosomes isolated from the serum of PD patients contain α-synuclein, with higher levels observed in late-stage patients [103]. PD plasma-derived exosomes also contain increased oligomeric and Ser129-phosphorylated α-synuclein [104]. Increased α-synuclein levels in plasma neuronal exosomes of early-stage PD patients have been linked to disease progression [105]. Recent research suggests that exosomes provide an environment for α-synuclein aggregation, mediating its propagation between neurons [69]. Injecting exosomes from PD patients into mice induces PD-like pathology, such as dopaminergic neuron degeneration [106]. Mice stereotaxically injected with PD patient plasma-derived exosomes showed neuronal uptake and spreading of α-synuclein to the cortex and substantia nigra [72]. In contrast, cerebrospinal fluid from PD patients shows decreased α-synuclein, while plasma exosomal levels are elevated, reinforcing exosomes’ role as PD biomarkers [107].

### 5.3. Amyotrophic Lateral Sclerosis

Amyotrophic Lateral Sclerosis (ALS) is a fatal neurodegenerative disorder with increasing prevalence [108]. ALS causes the degeneration of motor neurons, leading to muscle atrophy and death. Mutations in SOD1 and TARDBP (which encodes TDP-43) have been linked to ALS pathology [109]. In vitro studies suggest that exosomes aid in the propagation of misfolded SOD1. Mice overexpressing mutant SOD1 have significantly more exosomal SOD1 compared to non-transgenic mice [110]. Additionally, ALS patients show elevated exosomal TDP-43 levels over time, suggesting that exosomal TDP-43 may be a potential ALS biomarker [100,111].

### 5.4. Pediatric CNS Tumors

Childhood CNS tumors account for 25% of all pediatric neoplasms and are the primary cause of cancer-related deaths in children [112]. Among these, low-grade gliomas are the most frequent, classified by the World Health Organization (WHO) into four grades: grade I (pilocytic astrocytoma, PA), grade II (diffuse astrocytoma), grade III (anaplastic astrocytoma), and grade IV (glioblastoma multiforme) [113]. PA is one of the most common CNS tumors in pediatric patients, representing a complex clinical entity [114]. While PA can occur sporadically, it is also associated with neurofibromatosis type 1 in approximately 15% of cases, and is characterized by its predominantly benign nature, slow growth, and favorable prognosis following complete surgical resection [115]. A recent review by Bauman et al. (2022) provided further insights into pediatric PA. Maximal resection is the standard first-line therapy, as the extent of surgical removal significantly impacts recurrence-free survival [116]. The recent advancements in surgical techniques and adjuvant therapies resulted in improved 5-year survival rates, now approaching 75%, for children diagnosed with low-grade gliomas [117]. However, complete resection is not always achievable, particularly for deep midline supratentorial PAs, where the possibility of resection is dependent on anatomical location. Although 32% of PAs are primarily found in the posterior fossa, they can also arise in the cerebral cortex, optic pathways, hypothalamus, brainstem, and spinal cord. Approximately 90% of pediatric PAs exhibit genetic alterations in the RAS–mitogen-activated protein kinase (MAPK) pathway, primarily due to the activation of the v-Raf murine sarcoma viral oncogene homolog B (BRAF). Targeted therapies utilizing B-Raf inhibitors, such as vemurafenib and dabrafenib, have shown promise in preclinical models and clinical trials for BRAF-mutated PA forms [118,119]. The study by Kim et al. (2023), which compares the clinical characteristics and treatment outcomes of pediatric and adult PA patients, provides valuable data on age-related differences [120]. However, certain pediatric PAs demonstrate clinical variability, recurrence, and poor progression-free survival. Among these are non-BRAF-mutated PAs and those with increased mitotic activity and necrosis, known as PA with anaplasia (PA-A), which are not responsive to immunotherapy [121]. These non-BRAF-mutated and PA-A tumors lack specific therapeutic targets, and treatment remains reliant on chemotherapy, which is associated with significant morbidity and can adversely affect the long-term quality of life of patients [122]. Kinases play crucial roles in regulating cellular processes, including carcinogenesis [123]. Protein phosphorylation governs a wide range of biological functions [124]. Recent studies have identified additional kinase mutations beyond B-Raf, including those in the Raf-1 proto-oncogene (RAF1) and fibroblast growth factor receptor 1 (FGFR1) [125]. A comprehensive understanding of the expanding range of kinases implicated in pediatric PA is essential. Liquid biopsy techniques, which analyze circulating tumor DNA, RNA, cells, and EVs using high-throughput methods, have the potential to identify biomarkers and key signaling pathways [126]. A study highlighted the utility of DNA methylation profiling as a powerful tool for distinguishing between various pediatric brain tumors, including Pas [127]. Research on liquid biopsy in low-grade gliomas has been limited compared to that on other malignant brain tumors. The use of serum poses a significant challenge for identifying brain-specific protein markers due to the selective nature of the BBB [128]. In contrast, CSF is a promising source for biomarker discovery, as it is in direct contact with brain tissue and tumor masses and serves as a primary route for metastasis. Previous mass spectrometry studies have revealed that CSF contains numerous unique proteins, making it a valuable biochemical window into brain pathology [129]. Nevertheless, ethical and volume constraints limit CSF collection via lumbar puncture. Alternatively, CSF from extraventricular drainage (EVD) allows for large-volume and serial sampling. This unrestricted volume enables the isolation of EVs from EVD-derived CSF. Previous proteomic studies of EVD-derived CSF and its EVs demonstrated the ability to differentiate between pediatric brain tumor and non-tumor conditions, regardless of tumor type [130]. Moreover, bioinformatic analyses of these data from medulloblastoma (MB) patients and controls indicated that most potential disease biomarkers were localized within EVs and would be lost in unprocessed samples [131]. The comprehensive characterization of CSF-derived EVs from low-grade gliomas such as PA is still lacking. While diagnostic approaches primarily rely on histopathology and neuroimaging, there is a critical need for novel biomarkers to enhance therapeutic stratification, the detection of residual disease, and recurrence monitoring. The identification of reliable biomarkers could facilitate the development of non-invasive methods for monitoring therapeutic response and disease progression, thereby improving overall clinical outcomes [132,133].

## 6. The Potential Therapeutic Applications of Exosomes Derived from Stem Cells in the Treatment of Neurodegenerative Diseases

**Figure 6:**
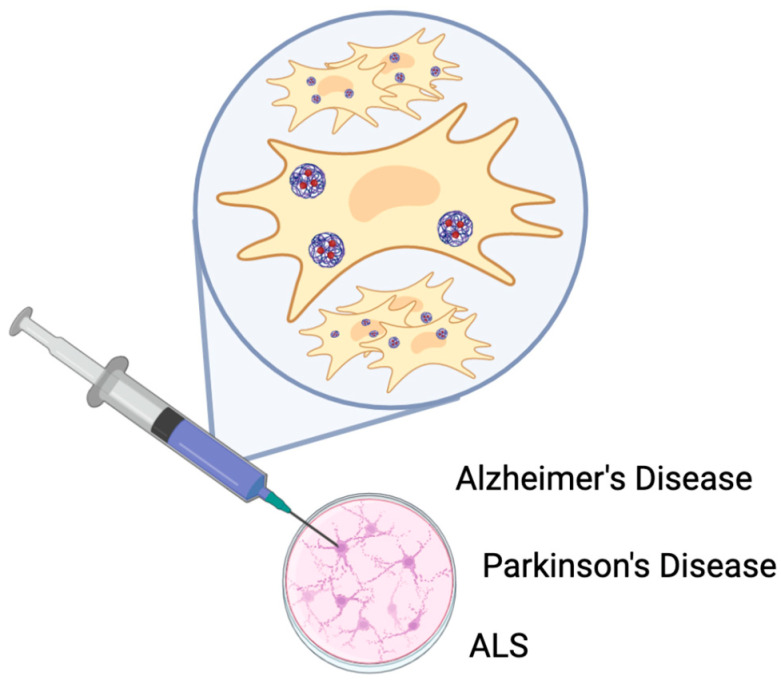
An illustration depicting the potential therapeutic applications of exosomes derived from stem cells in the treatment of neurodegenerative diseases. Exosomes, secreted by stem cells, carry bioactive molecules that can modulate cellular function. These exosomes can cross the blood–brain barrier, delivering therapeutic cargo to target cells in the brain, and potentially promoting neuroprotection and regeneration in conditions such as Alzheimer’s Disease, Parkinson’s Disease, and Amyotrophic Lateral Sclerosis (ALS).

The current treatments for neurodegenerative disorders offer limited therapeutic benefits, primarily providing symptomatic relief without halting or reversing disease progression [134]. Nevertheless, stem cell-derived exosomes are emerging as a potential therapy for these disorders due to their advantageous properties, as detailed in the following sections. Recent studies suggest that MSC-derived exosomes exhibit superior therapeutic effects compared to MSCs alone [135]. Exosomes can be stored for extended periods without the need for toxic preservatives [136]. The use of exosomes circumvents the risk of tumorigenesis since they do not undergo division. Exosomes can be administered via intranasal or intravenous routes [137]. One of the main challenges in developing new therapies for neurodegenerative diseases is crossing the BBB, as most small-molecule drugs cannot penetrate it. However, exosomes can efficiently cross the BBB due to their hydrophobicity and low water solubility. Once in the brain, exosomes retain their bioactive properties, further highlighting their potential as a therapeutic option for neurodegenerative disorders. Moreover, exosomes exhibit a long half-life and can be repeatedly administered systemically without evident toxicity, reinforcing their safety profile. In neurodegenerative conditions, where BBB integrity is compromised, exosomes could repair the damaged BBB [138]. Additionally, exosomes are biodegradable, with low immunogenicity and toxicity following systemic administration [137]. Due to their structure as lipid bilayer vesicles, exosomes present an attractive drug delivery system. Efforts have been made to enhance their therapeutic efficacy through genetic or chemical modification. For example, transfecting cells with specific miRNAs results in exosomes that overexpress these miRNAs, allowing them to silence genes in target cells [139]. Furthermore, since exosomes can be isolated from patients themselves, they can be modified and re-administered to the same donor, reducing immunogenicity and toxicity compared to manufactured exosomes [140]. This makes personalized exosome-based therapies particularly appealing, as exosome isolation and reinjection are minimally invasive (Figure 6). Exosomes have been identified in human biological samples, in order to find potential biomarkers useful for early diagnoses of neurological diseases such as miRNA miR-135a, miR-193b, and miR-385. Recently, several reports from cellular and animal models had pointed out the regulatory mechanisms involving exosomes in disease development, highlighting therapeutic possibilities and preclinical evidence of exosomes derived from various types of stem cells for exo-based therapy (E-MSCs, Embryonic Mesenchymal Stem Cells; BMSc, Bone Mesenchymal Stem Cell; ASC/ADSC, Adipose Tissue Mesenchymal Stem Cell) and also exploring their utility in exo-based nanomedicine for various neurological complications. The pathogenic proteins involved in neurodegenerative diseases are loaded into intraluminal vesicles (ILVs) of a late endosomal compartment, in the multivesicular bodies (MVBs), then subsequently secreted extracellulary via exosomes, which lack the capacity for cellular self-repair and contribute to spread their pathological content, among neurons. Endocytic alterations and abnormalities of MVB formation, commonly found in these diseases, suggest that impairment of exosome generation is associated with development, and with the intercellular spread of misfolded or toxic proteins in disease pathogenesis. In Table 3, we summarized human disease exosome-associated proteins involved in neurodegenerative diseases.

### 6.1. Stem Cell-Derived Exosomes for Treating Alzheimer’s Disease (AD)

While numerous preclinical studies have shown that exosomes are promising candidates for treating neurodegenerative diseases, their clinical application is hindered by the limited secretion of exosomes from stem cells. Typically, less than 1 µg of exosomal protein is yielded from 1 mL of culture media. Research indicates that cells subjected to stress conditions increase the production of multivesicular bodies and secrete more exosomes. One strategy to address the low yield is heat shocking (HS) the cells prior to isolation. A study found that neural stem cells exposed to 42 °C for 3 h produced significantly higher exosome concentrations and larger exosomes compared to non-heat-shocked cells. HS-derived exosome concentration was 13 times greater than non-HS exosomes. Despite having reduced protein diversity, HS-derived exosomes showed enhanced biological functions, such as the negative regulation of apoptosis and DNA damage, indicating their therapeutic potential. Moreover, cells treated with HS-derived exosomes demonstrated superior neuroprotection against hydrogen peroxide-induced cell death and Aβ-induced neurotoxicity compared to non-HS exosomes. HS-derived exosomes completely reversed Aβ-induced apoptosis and oxidative stress [141]. These findings suggest that HS treatment can enhance exosome production and cargo content while maintaining therapeutic efficacy. Another key feature of AD is neuroinflammation [142] and recent evidence suggests that exosomes exhibit anti-neuroinflammatory properties [143]. In an AD mouse model, the systemic injection of MSC-derived exosomes improved spatial learning and memory in the Morris Water Maze, decreased Aβ plaque load, and reduced activated microglia, supporting the anti-neuroinflammatory role of exosomes in AD [144]. Additionally, exosomes alleviate oxidative stress, another hallmark of neurodegenerative disorders [141,145].

### 6.2. Stem Cell-Derived Exosomes for Treating Parkinson’s Disease (PD)

In PD, exosomes loaded with dopamine have been shown to increase dopamine levels in the brain by over 15-fold. The administration of human umbilical cord MSCs in a PD mouse model improved PD-related behavioral symptoms, decreased neuronal apoptosis, and increased dopamine levels in the brain [146]. This study highlighted how exosomes in the blood can be loaded with dopamine, cross the BBB, and deliver dopamine to the substantia nigra and striatum, key regions affected in PD. Furthermore, MSC-derived exosomes alleviated cognitive impairment in a progressive PD model by influencing neuronal cholesterol metabolism [147]. Additionally, bone marrow MSC-derived exosomes demonstrated anti-inflammatory and antioxidative properties in a PD cell model [148]. Priming MSCs with α-synuclein has also been investigated as a strategy to enhance neuroprotection in PD. Pre-treatment of MSCs with α-synuclein was shown to enhance autophagy and stem cell stemness, conferring greater neuroprotection in dopaminergic neurons [149]. Increasing interest in engineered MSC-derived exosomes suggests their potential to provide targeted and more effective therapies for neurodegenerative diseases (Table 3).

### 6.3. Preclinical Studies on Stem Cell-Derived Exosomes in ALS

In ALS, exosomes from adipose stem cells have been shown to reduce SOD1 aggregation and mitochondrial dysfunction. Repeated injections of these exosomes improved motor neurons, reduced glial activation, and targeted lesion sites in an ALS mouse model [150]. Exosomes have also been found to reverse mitochondrial dysfunction in motoneuron cells expressing mutant SOD1(G93A) [151].

**Table 3:** Summarized human disease exosome-associated proteins involved in neurodegenerative diseases [152].

Human Disease	Exosome-Associated Protein	MVB/Endocytic Impairment
Creutzfeldt–Jakob disease	PrPc, PrPsc	MVB and endosome enlargement
Alzheimer’s Disese	Aβ	Endosome enlargement
	APP	Overexpression of RabGTPases
	BACE	CHMP2B high immunoreactivity
	Presenilin	PICALM, BIN1 mutation
AD and FTD	Tau	PICALM, BIN1 mutation
PD	α-Synuclein	CHMP2B mutation
	LRRK2	CHMP2B-positive inclusions
ALS	SOD1	CHMP2 mutation
	TDP-43	Axonal exosome transport deficits
Polyglutamine disease	Heat shock protein	
Huntington disease	HSP40, HSP70, HSP90	
Schizophrenia	Dysbindin-1B	Mutations in BLOC-1 subunits, and dysbindin, and muted

### 6.4. Clinical Trials Testing Exosomes for Neurodegenerative Disorders

While no exosome-based products have been approved by the FDA, the number of clinical trials investigating exosome-based therapies is rapidly increasing. As of 29 November 2022, 82 clinical trials using exosomes for various diseases were registered on ClinicalTrials.gov, with many focusing on respiratory diseases and cancer. Some trials are currently assessing the efficacy of exosomes in treating AD and PD (Table 4).

## 7. Discussion

**Figure 7:**
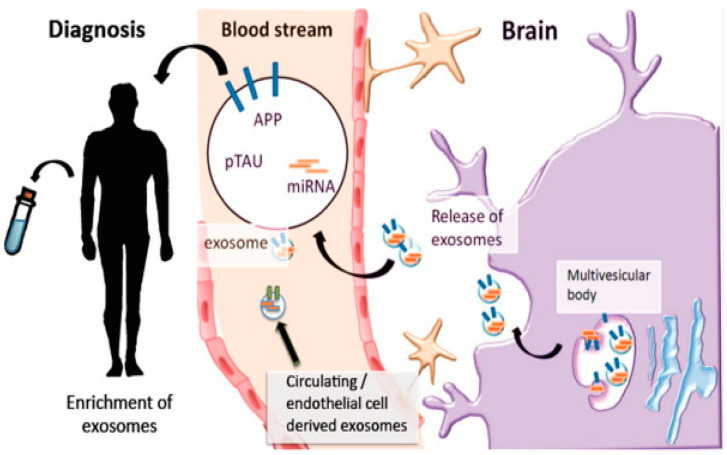
Exosomes as an innovative diagnostic tool for CNS disorders. Exosomes can be released by nearly all cell types. Exosomes released by brain cells can cross the BBB and can be detected in the bloodstream. Similarly, endothelial and peripheral cells also secrete exosomes into the circulation. These exosomes can be isolated from blood samples and used for detecting various proteins and nucleic acids. Additionally, exosomal membrane markers may be used to identify the cellular origin of the exosomes. Reprinted/adapted from ref. [154].

Exosomes are emerging as key mediators in intercellular communication, and their potential as diagnostic and therapeutic tools for neurological disorders is attracting increasing attention (Figure 7). Numerous experimental studies have demonstrated the promising diagnostic and therapeutic efficacy of exosomes in neurological conditions. However, whether exosomes are the most appropriate candidates for a diagnosis or therapy remains uncertain. Several challenges hinder their clinical application. One of the primary obstacles is the isolation and purification of exosomes. This challenge arises from their size and physicochemical properties, which often overlap with those of other nanoparticles such as lipoproteins, protein complexes, and chylomicrons. Ultracentrifugation, the most used method, is labor-intensive and inefficient for processing large sample volumes. Moreover, the density overlap between exosomes and high-density lipoproteins (HDL) in plasma or serum complicates their separation, as ultracentrifugation relies on density gradients. Immune-affinity capture techniques can isolate exosomes with specific surface proteins, but the yield is often low. Ultrafiltration fails to distinguish exosomes from chylomicrons and lipoproteins, while size exclusion chromatography encounters similar issues, with the added complication of requiring additional steps to reduce the large sample volumes collected. Microfluidics, though promising, involves complex material and technological requirements, making it impractical for large-scale sample handling.

It is crucial to develop a consistent and standardized approach for isolating exosomes with both high purity and high yield. Furthermore, MSCs provide therapeutic benefits through the combined action of MSC-derived exosomes and soluble factors. To eliminate the interference of soluble factors, Gao et al. analyzed the cytokines in a supernatant and found no soluble cytokines present. They discovered that the cytokines were associated with exosomes in an insoluble form [145]. To enhance the therapeutic potential of exosomes and improve their drug delivery capacity in vivo, bioengineered exosomes have been developed. These engineered vesicles can cross the BBB, exhibit low toxicity and immunogenicity, and offer promising drug delivery capabilities. Nevertheless, drug loading techniques, such as electroporation, freeze–thaw cycles, ultrasonic treatment, and extrusion, can compromise the integrity of the exosome membrane by creating transient pores. Therefore, optimal methods for drug loading into exosomes require further investigation. Despite their potential, our understanding of exosomes remains incomplete, and concerns about their clinical safety have yet to be fully addressed. It is crucial to further elucidate the mechanisms underlying exosome biogenesis, secretion, and transport to address these limitations and facilitate their clinical application. Future research should focus on ensuring the safety, efficacy, high yield, purity, and uniformity of exosomes during their translation to clinical settings.

In conclusion, future investigations into exosomes will not only enhance our understanding of their role in the pathogenesis of neurological disorders but also pave the way for novel approaches in the clinical diagnosis and treatment of these diseases. As a subset of extracellular vesicles (EVs), exosomes are anticipated to become innovative tools in the diagnosis and therapeutic management of neurological disorders.

## 8. Conclusions and Future Perspectives

A key challenge in exosome research is developing efficient methods for their separation and purification, given their small size and the wide variety of extracellular vesicles present in blood. The comparative analysis of exosomes from blood, spinal fluid, and brain tissue is of particular importance [155,156]. Although ultracentrifugation remains the standard method for exosome extraction, it is limited by the need for costly equipment and extensive processing time. Kit-based extraction techniques also encounter various difficulties. However, new methods are emerging, as demonstrated by recent studies [157].

Although exosomal research has gained significant momentum over the last decade, much remains unknown about these small extracellular vesicles and their role in disease. For example, enhancing exosomal yield without compromising their therapeutic function is critical for successful clinical trials. Additionally, the cargo of exosomes is important because it reflects the characteristics of their donor cells. A key question is why exosomes derived from the same cell line exhibit variability in diameter. Furthermore, understanding whether this size variability affects the contents of exosomes is essential. Future research should focus on determining how different exosomal cargo influences their therapeutic effectiveness. Moreover, improving the ability of exosomes to reach target organs is crucial for ensuring their delivery to disease sites upon systemic administration. Finally, it is essential to understand how exosomes selectively target specific tissues and how they are cleared from the body.

Exosomal research shows great promise as both a biomarker and a therapeutic tool for neurodegenerative disorders. Exosomes derived from MSCs have been widely studied and used in clinical trials, but exosomes from other types of stem cells, such as neural stem cells, also show promise in treating neurodegenerative diseases in cell and animal models. Despite these advances, many questions remain: how does the heterogeneity of isolated vesicles influence disease progression, and what are the long-term effects of exosome therapy? To enhance exosome yield and specificity, future research must address these issues, particularly in the treatment of various neurodegenerative diseases.

## Figures and Tables

**Table 1:** A comparative analysis of various exosome isolation techniques. Reprinted/Adapted from ref. [29].

ISOLATION TECHNOLOGY	SEPARATION PRINCIPLE	SAMPLE SIZE	ADVANTAGES	DISADVANTAGES
***Ultracentrifugation***	Molecular size, density, and shape	Large	Low risk of pollution, low reagent cost	Expensive equipment, time-consuming, poor biological activity, and integrity of exosomes
***Size exclusion chromatography***	Molecular size	Medium	Yield, purity, integrity, and biological activity of exosomes can be ensured	Special equipment
***Immune-affinity capture***	Specific binding of antigen and antibody	Small	High purity	High cost, low yield
***Ultrafiltration***	Molecular size and shape	Large	Efficient and convenient	Low purity, exosomes may partially remain on the membrane
***Microfluidic***	Immune affinity, size, and density	Small	Fast, low cost, convenient, and automated	The selectivity and specificity need to be verified

**Table 2:** A summary of the known functions of exosomes in the CNS.

Function	Details	Examples
Intercellular Communication	Facilitate exchange of proteins, lipids, RNA, and other molecules between CNS cells.	Neuron–glia signaling, astrocyte support.
CNS Development	Contribute to synapse formation, neuronal differentiation, and neural circuit organization.	Role in neurogenesis during brain development.
Homeostasis Maintenance	Regulate cellulular processes like waste clarance and immune response.	Clearing misfolded proteins via microglial exosomes.
Pathological Progression	Promote or inhibit disease progression depending on cargo and context.	Spread of toxic proteins in Alzheimer’s and Parkinson’s Diseases.
Biomarker Potential	Detectable in peripheral blood, providing non-invasive diagnostic tools for CNS disorders.	Exosomes carrying amyloid-beta or alpha-synuclein.
Therapeutic Potential	Deliver neuroprotective molecules and modulate inflammation or repair mechanisms.	MSC-derived exosomes for stroke recovery.

**Table 4:** Clinical trials on exosomes for the management of neurological diseases/disorders [153].

Clinical Trial No. (CT)	Phase	Trial Name	Pathological Condition	Intervention
NCT05370105	1	EVs as Stroke Biomarkers (EXO4STROKE)	Stroke	Blood withdrawal
NCT01811381	2	Curcumin and yoga therapy for those at risk for AD	AD	Drug: Curcumin Behavior: Aerobic yoga Behavior: Non-aerobic yoga
NCT05490173	Not applicable	Long-term Regular Tai Chi Training for Healthy Elderly Circulating EXOs Release and Cognitive Neural Circuits/Networks Activity Characteristics Research	Cognitive	Long-term irregular exercise group
ChiCTR2200057303	Retrospective study	A single-center randomized controlled study of human neural stem cell-derived EXOs in the treatment of ischemic stroke	Ischemic stroke	Treatment group: EXOs Control group: Saline
ChiCTR2100048661	Retrospective study	Differential diagnosis of unipolar depression and bipolar depression based on neurogenic exosome miRNA	Depression	Gold standard: Hamilton Depression Scale—17 items, Young’s Manic Scale, DSM-5, M.I.N.I scale; index test: Methods—Neurogenic EXOs were isolated and miRNA was sequenced; biomarker: Neurogenic exosome miRNA; equipment: Illumina MiSeq
ChiCTR2100044323	1	EXOs alterations following electroconvulsive therapy in depression	Depression	Depression cases: Electroconvulsive therapy
ChiCTR2000039377	1	EXOs derived from Neural stem cell Induces Osteogenesis and angiogenesis following traumatic brain injury	TBI	Healthy patient group: Nil; patient with limb fracture only: Nil; patient with TBI only: Nil; patient with limb fracture following TBI: Nil
ChiCTR2000038262	Retrospective study	The effects on circRNAs’ expression in the plasma EXOs of patients with Perioperative Neurocognitive Disorders after noncardiac surgery	Cognitive disorders	Control group: After induction of anesthesia, 0.9%NS was injected under load, and then 0.9%NS was continuously pumped into the suture; trial group: After anesthesia induction, 0.25 mg/kg S-ketamine was injected under load, and 0.125 mg/kg/h S-ketamine was continuously pumped until the suture
ChiCTR2000032579	Retrospective study	The Safety and the Efficacy Evaluation of Allogenic Adipose MSC-Exos in Patients with AD	AD	Low-dose group: 5 μg MSC-Exos administrated for nasal drip; mid-dose group: 10 μg MSC-Exos administrated for nasal drip; high-dose group: 20 μg MSC-Exos administrated for nasal drip
NCT04202770	1	Focused Ultrasound and EXOs to Treat Depression, Anxiety, and Dementias	Anxiety and dementia	EXOs
ChiCTR1900026776	1	Screening for early diagnosis biomarkers of mental disorders in serum EXOs	Mental disorders	N/A
NCT05886205	1	Induced Pluripotent Stem Cell Derived EXOs Nasal Drops for the Treatment of Refractory Focal Epilepsy	Epilepsy	Drug: iPSC-Exos
ChiCTR2200064447	Retrospective study	Study on the mechanism of exosome miRNA mediated autophagy in temporal lobe epilepsy	Epilepsy	Oxcarbazepine group: Take oxcarbazepine; oxcarbazepine + CLMD group: Take oxcarbazepine + CLMD; control group: None
